# Asthma data in Türkiye between 2016 and 2022: a nationwide descriptive and observational study covering the entire adult population

**DOI:** 10.55730/1300-0144.6185

**Published:** 2026-02-09

**Authors:** Kurtuluş AKSU, Gürgün Tuğçe VURAL SOLAK, Naim ATA, Mustafa Hamidullah TÜRKKANI, Mustafa Mahir ÜLGÜ, Şuayip BİRİNCİ, Funda AKSU

**Affiliations:** 1 Division of Immunology and Allergy, Department of Chest Diseases, Ankara Atatürk Sanatoryum Training and Research Hospital, University of Health Sciences, Ankara, Turkiye; 2 Ministry of Health, Ankara, Turkiye; 3 Department of Chest Diseases, Ankara Etimesgut Şehit Sait Ertürk State Hospital, Ankara, Turkiye; 4 Department of Chest Diseases, Ankara Atatürk Sanatoryum Training and Research Hospital, University of Health Sciences, Ankara, Turkiye

**Keywords:** Asthma, asthma hospitalization, asthma treatment, inhaler treatment, inhaler device, comorbidities of asthma, nebulizer use, montelukast

## Abstract

**Background/aim:**

Determining the current burden of asthma in countries is important in the diagnosis and follow-up of asthma patients and for the development of national health policies. Our study aimed to clarify the epidemiological and clinical data regarding asthmatic adults in Türkiye.

**Materials and methods:**

The study was designed as a nationwide descriptive, observational cross-sectional study. Data on adult patients who were followed with the diagnosis of asthma in all public, private, and university hospitals in Türkiye between January 2016 and December 2022 were obtained from the e-Nabız database of the Ministry of Health and analyzed.

**Results:**

The number of patients followed with a diagnosis of asthma in Türkiye between 2016 and 2022 was 2,700,183. The mean age of these patients was 45.70 years (standard deviation: 15.87) and 74.5% of them were female. Of the patients, 92.3% were prescribed dry powder inhalers, 98.41% metered dose inhalers, and 80.38% montelukast. The short-acting beta-2 agonist (SABA) prescription rate was 65.74% and SABA was prescribed as the sole medication for 0.10% of patients. The percentage of patients prescribed nebulized treatment was found to be 69.83%. Among asthmatic adults, the rate of at least one emergency room visit was 12.4%, the rate of hospitalization in the ward was 5.6%, and the rate of intensive care unit admission was 0.1%.

**Conclusion:**

Asthma is a major health burden in Türkiye, as is the case around the world. National planning for the diagnosis and follow-up of patients needs to be improved and continued.

## 1. Introduction

Asthma is a disease characterized by chronic airway inflammation and variable expiratory airflow limitation, accompanied by respiratory symptoms such as wheezing, cough, shortness of breath, and chest tightness. The severity and persistence of symptoms may vary during the course of asthma. This variable nature of asthma plays a key role in distinguishing the disease from other respiratory diseases. Symptoms and airflow limitation may sometimes resolve spontaneously or with treatment, but patients may also experience exacerbations, which can be life-threatening. Treatment goals include the control of symptoms, enabling asthmatic individuals to continue their daily activities, preventing exacerbations, improving quality of life, decreasing mortality rates, and minimizing medication side effects [1]. The frequency of asthma, which affects approximately 300 million people worldwide, may vary between countries and even between regions within countries. The prevalence of asthma in different societies is reported to vary between 1% and 29%. However, our current knowledge is not sufficient to explain the reason for these differences [2–4]. Recent studies have reported a global prevalence of asthma ranging between 2% and 3% in children and adolescents and 6% and 7% in adults. However, there are also studies indicating that the prevalence of asthma in adults is 2%–3% in some low-income countries and approximately 10% in high-income countries [5,6].

Limited data from epidemiological studies conducted in different provinces of Türkiye indicate that the prevalence of asthma is between 1.2% and 9.4% [7–14]. The most comprehensive study conducted to date to determine the prevalence of asthma in Türkiye was the Allergy Prevalence and Risk Factors (PARFAIT) study, which was a cross-sectional and survey-based study implemented in 14 cities. This study revealed that the prevalence of asthma was 7.1% in men and 9% in women [15]. The main problem in prevalence studies of asthma stems from the fact that the criteria and study methods used in different studies are not the same.

The cost of asthma-related healthcare around the world has increased over the years. For example, this cost was 53 billion dollars in the United States in 2007, and it increased to 82 billion dollars in 2013 [16]. Thus, the cost of asthma is significant in relation to patient care, emergency room visits, and severe persistent asthma, as well as reduced working capacity and disability due to the disease [17–19].

Clarifying the current status of asthma patients in countries is important in determining the current burden of asthma and developing national health policies. The present study aims to determine the current status of asthma in Türkiye by determining clinical characteristics such as the number of asthmatic adults, their treatments and hospitalizations, and the distribution of patients across different regions.

## 2. Materials and methods

### 2.1. Data collection

The national descriptive and observational cross-sectional design of this study was approved and implemented in accordance with the Ministry of Health (MoH) of Türkiye (Approval Number: 95741342-020). The design and procedure of the study were in accordance with the Declaration of Helsinki. Furthermore, the study was prepared and reported in accordance with the Strengthening Reporting of Observational Studies in Epidemiology (STROBE) guidelines.

This study used anonymized data from the MoH’s national electronic e-Nabız database. At the time of this research, the database included the health records of over 85 million citizens from January 1, 2016, through December 31, 2022. The Turkish MoH has been overseeing this national electronic database, which merged all other databases and includes information on procedures, laboratory results, medications, and deaths, since 2016. It is officially required for all hospitals to report such data via this system since 2016. Since the Turkish MoH governs this system and merged all previous databases, the data of patients followed with a diagnosis of asthma in public, private, and university hospitals between January 1, 2016, and December 31, 2022, were obtained from the e-Nabiz database of the MoH.

### 2.2. Study population

Codes from the 10th revision of the International Classification of Diseases (ICD-10) related to asthma were screened, including J45 (Asthma), J45.0 (Asthma, allergic), J45.1 (Asthma, intrinsic), J45.8 (Asthma, mixed), J45.9 (Asthma, unspecified), and J46 (Status asthmaticus). Individuals aged 18 and over who received one of the specified asthma-related ICD-10 codes in departments of family medicine, internal medicine, chest diseases, and allergy immunology at least twice between January 2016 and December 2022 and who were prescribed inhaler treatment with these codes at least once were included in the study.

Patients whose medical records contained any of the ICD-10 codes related to chronic obstructive pulmonary disease (COPD) (J44: COPD, other; J44.0: COPD with acute lower respiratory tract infection; J44.1: COPD with acute exacerbations, unspecified; J44.8: COPD, other, unspecified; J44.9: COPD, unspecified), or bronchiectasis (J47) were excluded.

Data on age, sex, geographical region of residence, annual number of emergency and outpatient clinic admissions, frequency and duration of hospitalization in the ward or intensive care unit (ICU), comorbid chronic diseases based on ICD-10 codes, and pregnancy status were obtained for all patients.

Patients diagnosed with asthma between 2016 and 2022 with at least one record of a chronic disease according to ICD-10 codes (diabetes mellitus [E10, E11, E12, E13, E14], hypertension [I10, I15], coronary artery disease [I24, I25], heart failure [I50], cerebrovascular disease [G46, I61, I62, I64], myocardial infarction [I21, I22, I23], gastrointestinal system diseases [K50, K51, K58, K59, K21], renal failure [N17, N18, N19], obesity [E66], nasal polyps [J33], sleep apnea [G47.3], rhinitis [J30, J31], and allergic rhinitis [J30.1, J30.2, J30.3, J30.4]) were also noted.

The asthma treatments used by the patients were recorded according to Anatomical Therapeutic Chemical Classification codes and then grouped as metered dose inhalers (MDIs), dry powder inhaler (DPIs), nebulizer drugs, leukotriene modifiers, long-acting muscarinic antagonists (LAMAs), and biologics (omalizumab and mepolizumab). The active substances in the MDI group were salbutamol, salbutamol-ipratropium, and levosalbutamol-ipratropium, which are asthma reliever medications; budesonide, ciclesonide, and fluticasone, which are inhaled corticosteroids (ICSs); formoterol-beclomethasone, formoterol-budesonide, formoterol-fluticasone, formoterol-mometasone, and salmeterol-fluticasone, which are combinations of ICSs and long-acting beta-2 agonist (LABA) medications; and the formoterol-glycopyrronium-beclomethasone combination, which is a fixed-dose ICS/LAMA/LABA combination. The active ingredients in the DPI group were budesonide and fluticasone, which are ICSs, and fluticasone-vilanterol, formoterol-beclomethasone, formoterol-budesonide, formoterol-fluticasone, and salmeterol-fluticasone, which are ICS-LABA combinations. The active ingredients in the nebulizer drug groups were budesonide and fluticasone as ICSs and salbutamol and salbutamol-ipratropium as asthma reliever medications. The active ingredient of the leukotriene modifiers was montelukast. The LAMA active ingredient was tiotropium. It was determined that these drugs had been prescribed at least once for the analyzed patients.

To assess short-acting beta-2 agonist (SABA) use, the prescription rate of MDIs containing salbutamol, salbutamol-ipratropium, and levosalbutamol-ipratropium combinations was analyzed separately.

### 2.3. Statistical analysis

IBM SPSS Statistics 22 and Microsoft Excel 2016 were used for statistical analysis. Frequency analyses were performed for categorical variables and the results were given as numbers and percentages. Numerical values were expressed as mean and standard deviation values.

### 2.4. Ethical declaration

The study protocol was approved by the Turkish MoH and conducted in accordance with approval number 95741342-020. This study used anonymized data from the Turkish MoH’s national electronic database. The design and procedure of the study were in accordance with the Declaration of Helsinki.

## 3. Results

### 3.1. Demographic and epidemiological data

**Figure 1:**
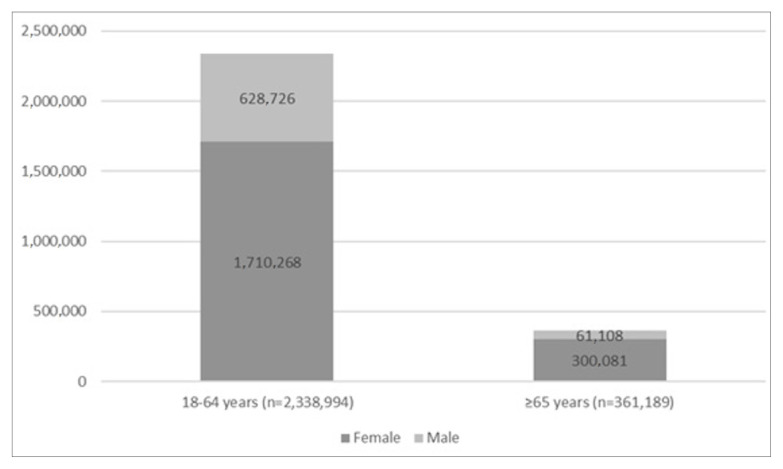
Chart of asthma patient distributions according to age range and sex.

**Figure 2:**
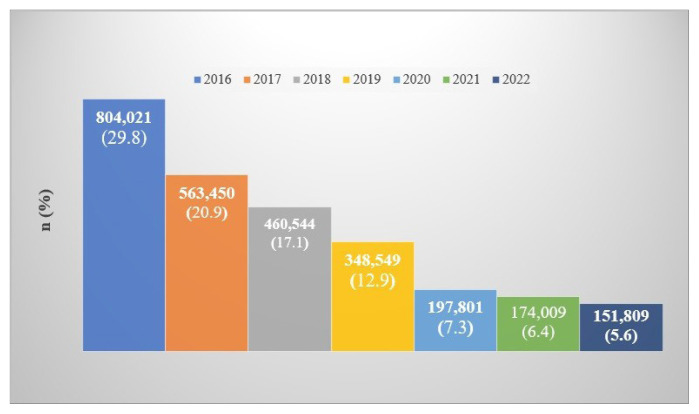
Numbers of patients diagnosed with asthma according to years.

It was found that a total of 2,700,183 patients were followed with a diagnosis of asthma in Türkiye between January 2016 and December 2022. Their mean age was 45.70 ± 15.87 years and the sex distribution was in favor of female patients at a total of 2,010,349 (74.5%) (Table 1). As shown in Figure 1, 2,338,994 (86.6%) patients were in the age group of 18–64 years and 361,189 (13.4%) were ≥65 years. Furthermore, according to the national database (Figure 1), 804,021 patients were diagnosed with asthma in 2016. However, since data from years before 2016 were not available due to the study design, it is not known how many of these patients were newly diagnosed with asthma in 2016. For the following years, the number of patients newly diagnosed with asthma was 563,450 in 2017, 460,544 in 2018, 348,549 in 2019, 197,801 in 2020, 174,009 in 2021, and 151,809 in 2022 (Figure 2). The incidence of asthma per 100,000 people was found to be 697 for 2017, 562 for 2018, 419 for 2019, 237 for 2020, 205 for 2021, and 178 for 2022. Turkish Statistical Institute data were used for the annual population of Türkiye.

### 3.2. Distribution of asthma patients by geographical regions

**Figure 3:**
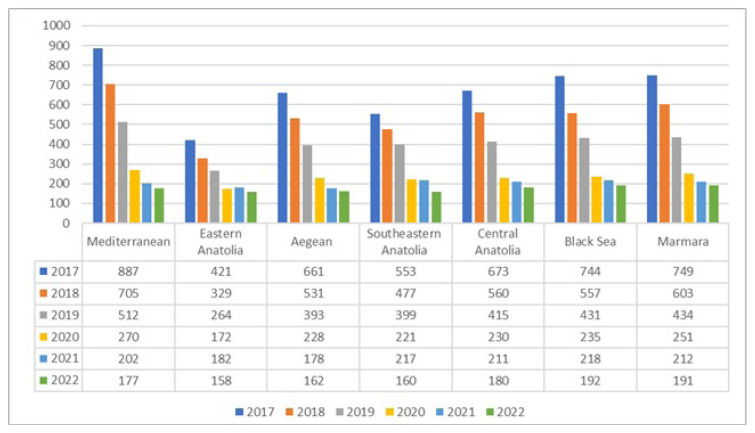
Distribution of asthma incidence by geographic region and year for the period of 2017–2022.

According to the distribution of asthma patients by geographical regions, the most asthma patients were followed in the Marmara Region (852,550; 31.57%) and the fewest asthma patients were followed in the Eastern Anatolia Region (124,531; 4.61%). The distribution of asthma patients according to geographical regions and years in Türkiye is shown in Table 2. The distribution of asthma incidence by geographic region and year for the years of 2017–2022 is shown in Figure 3.

### 3.3. Data on health institutions following asthma patients and the health insurance of these patients

It was determined that 2,155,833 (79.8%) of the patients diagnosed with asthma were followed in public institutions affiliated with the MoH, 86,165 (3.2%) in health institutions affiliated with universities, and 458,196 (17%) in private healthcare institutions. The distribution of the health insurance of all asthma patients is shown in Table 3.

### 3.4. Comorbidities of asthma patients

The comorbidities of asthma patients according to ICD-10 codes are listed in Table 4.

### 3.5. Pregnancy status of asthma patients

Pregnancy was detected in 246,070 (12.2%) of 2,010,349 female asthma patients between 2016 and 2022.

### 3.6. Medications of asthma patients

The rate of patients prescribed DPI for asthma treatment was 92.3%, the rate of MDI prescription was 98.41%, and the rate of montelukast prescription was 80.38%. The rate of SABA prescription was 65.74%. SABA was prescribed in combination with at least one of the main control treatments to 65.65% of the patients, while SABA was prescribed as the sole medication to 0.10%. The rate of patients prescribed nebulized treatment was found to be 69.83%. The rate of asthma patients prescribed inhaled tiotropium was 1.1%. Regarding biologic treatments for asthma patients, 0.43% of the study population received omalizumab and 0.02% received mepolizumab.

### 3.7. Outpatient, emergency room, ward, and intensive care admissions

Our analysis revealed that 334,256 (12.4%) of all asthma patients applied to the emergency department at least once. Regarding hospitalization rates among all asthma patients, 149,868 (5.6%) had at least one ward admission and 3764 (0.1%) had at least one ICU admission.

## 4. Discussion

This study has investigated the data of asthma patients in the general adult population in Türkiye based on the country’s electronic health database. With this nationwide cross-sectional study, the demographic characteristics of the asthmatic population, their comorbidities, medications used for asthma, frequency of outpatient and emergency department visits, and hospitalization rates were assessed. It was determined that 2,700,183 adults were followed with a diagnosis of asthma in Türkiye between January 2016 and December 2022.

The baseline sociodemographic characteristics of asthma patients revealed in this study are consistent with the epidemiological data worldwide. The average age of the patients was 45.70 ± 15.87 years and approximately three-quarters were female. The proportion of individuals with asthma over the age of 65 years is 13.4% in this country, reflecting the decreasing rate of asthma with older age. According to data published in Türkiye in 2013, the age-standardized asthma prevalence is 2.8% in men and 6.2% in women. Generally speaking, in all age groups, the prevalence of asthma diagnosed by a doctor is higher in women than in men, and women between the ages of 45 and 64 years are approximately three times more likely to have asthma than men [20]. Furthermore, 2018 data revealed that the adjusted prevalence of asthma in the general adult population over the age of 18 in Türkiye was 4.4% and the prevalence among women was twice as high as that for men [21]. Therefore, there does not seem to be much difference in asthma epidemiology over the years. However, in comparisons of historical data, some differences in the geographical distribution of asthma emerge. In 2018 it was reported that there was no significant difference in asthma prevalence across the regions of Türkiye [21]. However, according to the results of the present study, asthma patients are most common in the Marmara region and least common in the Eastern Anatolia region.

In the Global Health Data Exchange database, data from 195 different countries were collected, and the asthma incidence in 2017 was determined to be 0.56% [22]. In our dataset, the asthma incidence in 2017 was determined to be 697 per 100,000 people. In addition, according to 2019 data from the Global Burden of Disease Study, which included 204 countries worldwide and evaluated the incidence of asthma between 1990 and 2019, the incidence of asthma decreased from 601.20 per 100,000 people in 1990 to 477.92 in 2019 [23]. Similarly, in our study, we observed a decrease in the incidence of asthma over the years. The common point shown by these studies is that even though the incidence of asthma has decreased over the years, cases are still numerically rising.

Chronic accompanying health problems are more common in asthmatic individuals than in the normal population. According to 2018 data from Türkiye, 38.4% of asthma patients have chronic health problems. The most common comorbidity was cardiovascular disease [21]. In the present study, rhinitis and gastrointestinal comorbidities were the most common comorbidities in asthma patients. Asthma control could not be directly assessed with the dataset used in this study, but it is well known that gastrointestinal comorbidities and rhinitis increase disease burden, increase healthcare utilization, and impair asthma control. Therefore, asking asthma patients questions about these comorbid conditions and conducting further investigations when necessary may facilitate asthma control. The results of the present study also showed that cardiovascular diseases are seen at significantly high rates in adults with asthma, in line with previous findings. It was observed that 53% of asthma patients also had hypertension, and this rate is much higher than the hypertension prevalence rate of 31.2% reported for the general population of Türkiye [24]. The rate of diabetes among asthmatic individuals was also high in this study; up to 37% of asthma patients have diabetes in contrast to the diabetes prevalence of 11.1% reported for the general adult population in Türkiye [25]. According to the data analyzed in the present study, coronary artery disease and heart failure were present at rates of 25.3% and 6.2% among asthma patients. Thus, this study has provided important data on comorbidities associated with asthma. However, it should be noted that these diagnoses are based on ICD-10 codes assigned by healthcare providers and may not be related to active disease or disease severity. We did not include patients diagnosed with COPD in this study in order to better define the patient population, taking into account the prescribing patterns of healthcare providers. Consequently, we were unable to assess the presence of asthma–COPD overlap in elderly patients in particular.

Due to the structure of Türkiye’s healthcare system, almost all adult asthma patients have health insurance. The percentage of adult asthma patients without health insurance is less than 1%. The majority of adult asthma patients were followed in public institutions affiliated with the MoH, and a smaller portion were followed in private healthcare institutions. Therefore, patients appear to be able to easily access the healthcare services they need. However, despite easy access to health centers, the burden of asthma found in this study was still high, with 5.6% of asthma patients requiring ward admission. Furthermore, 0.1% of all asthma patients were admitted to the ICU at least once. These data also suggest that the desired level of control is not being achieved despite current treatment options for asthma.

Overreliance on reliever medications rather than controller medications in asthma treatment is a major cause of uncontrolled disease, serious exacerbations, and even death. The dispensing of 3 or more 200-dose SABA canisters per year is associated with increased risk of exacerbations, and dispensing of 12 or more canisters per year for a patient is associated with a markedly increased risk of death. These risks are higher with nebulized SABA [26,27]. In this context, it is a positive finding that the data analyzed in this study show that SABA treatment is not prescribed alone; rather, it is prescribed as a rescue treatment along with controller treatments. It was seen that 65.65% of the patients were prescribed SABA treatment along with at least one asthma-controlling treatment, and only 0.10% were prescribed SABA treatment alone. These SABA prescription rates are still very high, but the information obtained from this dataset does not include the number of SABA units prescribed or a patient’s SABA usage status. Therefore, we cannot establish a relationship between increased SABA usage and disease exacerbation and mortality. This should be taken into account when evaluating prescription status. The rate of prescribing nebulizer treatment to asthma patients was found to be as high as 70%. However, the high rate of nebulizer treatment prescriptions should be interpreted with caution. The data reflect at least one prescription, not necessarily regular or long-term nebulizer use. Nebulizer treatment being prescribed during asthma exacerbations and the easy access to nebulizer treatment by both healthcare providers and patients may have contributed to this high rate. In Türkiye, the rate of montelukast prescription for adult asthma patients was found to be as high as 80%. The high percentage of montelukast prescription may be related to the presence of allergic rhinitis, which frequently accompanies asthma. It should also be noted that prescriptions issued to patients do not necessarily reflect full treatment compliance and regular use. The rate of inhaled tiotropium prescription was only 1.1%.

The strength of this study is that it is not a survey study based on individual statements but rather a study based on data from physician-diagnosed asthma patients in the country’s electronic health database.

The main limitation of this study is that since it is not a prospective study, risk factors that adversely affect asthma control could not be examined. In addition, patient data have only been added to the database since 2016. The data presented for 2016 constitute the cumulative data of patients diagnosed with asthma prior to 2016 and those diagnosed with asthma in that year. This methodological feature should be taken into account when interpreting the findings. To limit asthma diagnoses as much as possible, we included only patients with at least two asthma diagnoses and at least one inhaler prescription. However, this approach, based on healthcare provider reports, may have led to overestimation or underestimation of asthma cases due to variations in daily practice. This should be taken into account when interpreting the national data. Finally, results concerning mortality rates were not analyzed due to concerns that the causes of death may not have been accurately reflected in the national health database.

In conclusion, this study evaluated the number of asthma patients and their geographical distribution, clinical features, treatments, and follow-up based on 7 years of data in Türkiye. The study has revealed that asthma is still a widespread health problem and health burden in this country.

## Figures and Tables

**Table 1:** Demographic features of asthma patients (n = 2,700,183).

Age (years) (mean ± SD)	45.70 ± 15.87
Sex Female Male	2,010,349 (74.5) 689,834 (25.5)

Data are expressed as n (%) unless otherwise stated. SD: Standard deviation.

**Table 2:** Distribution of asthma patients by geographical regions in Türkiye (n = 2,700,183).

Geographical regions	Asthma cases, n (%)	(%)*
Mediterranean	425,740 (15.77)	15.77
Eastern Anatolia	124,531 (4.61)	4.61
Aegean	328,764 (12.18)	12.18
Southeastern Anatolia	255,259 (9.45)	9.45
Central Anatolia	434,531 (16.09)	16.09
Black Sea	278,808 (10.33)	10.33
Marmara	852,550 (31.57)	31.57
Total	2,700,183	100

* Percentage ratios were calculated using the total number of adult asthma cases recorded throughout the entire study period (2016–2022) as the denominator.

**Table 3:** Distribution of health insurance types of asthma patients (n = 2,700,183).

Health insurance	n (%)
General health insurance scheme	2,127,449 (78.8)
Private insurance	33,556 (1.2)
Unknown	448,624 (16.6)
None	90,554 (0.4)

**Table 4:** Comorbidities of asthma patients (n = 2,700,183).

Comorbid chronic diseases	n (%)
Diabetes mellitus	999,196 (37)
Hypertension	1,432,374 (53)
Coronary artery disease	683,301 (25.3)
Heart failure	168,712 (6.2)
Cerebrovascular disease	119,722 (4.4)
Myocardial infarction	131,409 (4.9)
Gastrointestinal system diseases	2,296,977 (85.1)
Renal failure	115,678 (4.3)
Obesity	164,373 (6.1)
Nasal polyp	44,867 (1.7)
Sleep apnea	29,565 (1.1)
Rhinitis	2,285,324 (84.6)
Allergic rhinitis	1,257,841 (46.6)

## Data Availability

The dataset used and/or analyzed during the present study is available upon reasonable request.
